# Development and validation of a novel nomogram predicting clinically significant prostate cancer in biopsy‐naive men based on multi‐institutional analysis

**DOI:** 10.1002/cam4.6750

**Published:** 2023-11-28

**Authors:** Qingyu Ge, Sicong Zhang, Hewei Xu, Junjie Zhang, Zongyao Fan, Weilong Li, Deyun Shen, Jun Xiao, Zhongqing Wei

**Affiliations:** ^1^ Department of Urology The Second Affiliated Hospital of Nanjing Medical University Nanjing Jiangsu China; ^2^ Department of Urology The Second Clinical Medical College of Nanjing Medical University Nanjing Jiangsu China; ^3^ Department of Urology The First Affiliated Hospital of USTC, University of Science and Technology of China Hefei Anhui China

**Keywords:** multiparametric magnetic resonance imaging, nomogram, prostate‐specific antigen density, prostatic neoplasms

## Abstract

**Background:**

Prediction of clinically significant prostate cancer (csPCa) is essential to select biopsy‐naive patients for prostate biopsy. This study was to develop and validate a nomogram based on clinicodemographic parameters and exclude csPCa using prostate‐specific antigen density (PSAD) stratification.

**Methods:**

Independent predictors were determined via univariate and multivariate logistic analysis and adopted for developing a predictive nomogram, which was assessed in terms of discrimination, calibration, and net benefit. Different PSAD thresholds were used for deciding immediate biopsies in patients with Prostate Imaging‐Reporting and Data System (PI‐RADS) 3 lesions.

**Results:**

A total of 932 consecutive patients who underwent ultrasound‐guided transperineal cognitive biopsy were enrolled in our study. In the development cohort, age (odds ratio [OR], 1.075; 95% confidence interval [CI], 1.036–1.114), PSAD (OR, 6.003; 95% CI, 2.826–12.751), and PI‐RADS (OR, 3.419; 95% CI, 2.453–4.766) were significant predictors for csPCa. On internal and external validation, this nomogram showed high areas under the curve of 0.943, 0.922, and 0.897, and low Brier scores of 0.092, 0.102, and 0.133 and insignificant unreliability tests of 0.713, 0.490, and 0.859, respectively. Decision curve analysis revealed this model could markedly improve clinical net benefit. The probability of excluding csPCa was 98.51% in patients with PI‐RADS 3 lesions and PSAD <0.2 ng/ml^2^.

**Conclusion:**

This novel nomogram including age, PSAD, and PI‐RADS could be applied to accurately predict csPCa, and 44.08% of patients with equivocal imaging findings plus PSAD <0.2 ng/ml^2^ could safely forgo biopsy.

## 1 INTRODUCTION

Prostate cancer (PCa) is one of the most commonly genitourinary malignancies and the second leading cause of cancer‐related death in men worldwide, with an estimated 288,300 newly diagnosed cases and 34,700 males in the United States will die from PCa in 2023.^1^ In China, the incidence rate of PCa experienced an incredible 95.2% rise from 1990 to 2019, compared to a 13.2% growth globally over the same period.^2^, ^3^ Although no consensus exists on whether serum prostate‐specific antigen (PSA) screening should be extensively recommended, the majority of Chinese urologists prefer to execute this screening program due to its convenience and low cost but may induce serious harm from overdiagnosis and/or overtreatment.^4^ Actually, the primary objective of the screening test is to identify a clinically significant phenotype, namely clinically significant prostate cancer (csPCa) in prostatic carcinoma, and subsequently trigger pathological diagnosis with a biopsy.^5^ To achieve the early detection of csPCa, clinicians have been committed to developing the imageology, biomarkers, and technique of prostate biopsy for decades.

Multiparametric magnetic resonance imaging (mpMRI) is a noninvasive and mainstream diagnostic tool for csPCa, which is analogous to low‐dose computed tomography for lung cancer or mammography for breast cancer. The Prostate Imaging‐Reporting and Data System (PI‐RADS) based on mpMRI was generated to standardize imaging reports with five grades^6^, ^7^ and evidence is accumulating that the application of mpMRI plus PI‐RADS in biopsy‐naive patients could significantly augment negative predictive value (NPV) and improve diagnostic performance.^8^, ^9^ However, some studies found that a small proportion of patients with csPCa would be missed owing to the negative MRI findings,^10^, ^11^ so relying only on imaging findings is insufficient to obtain a satisfactory positive rate of prostate biopsy due to subjectivity and low specificity, which requires clinicians to weigh other information when making a clinical decision, such as age, digital rectal examination (DRE), PSA, and PSA related derivatives.

A randomized study revealed that the sensitivity of PSA for detecting PCa was only 40.4% with the 4.1 ng/mL cutoff and the specificity could also be affected by benign prostate hyperplasia, prostatitis, and ejaculation.^12^ PSA density (PSAD) is the combination of PSA and prostate volume (PV) and previous studies found that PSAD was a significant predictor of csPCa, especially in patients with gray‐zone PSA levels or ambiguous mpMRI.^13^, ^14^ Though guidelines have approved the implementation of some risk calculators and prediction models based on Western men in biopsy decision‐making, these models might create evident miscalibration for Chinese population.^15^ Additionally, earlier investigators included patients with prior negative transrectal ultrasound biopsies in their prediction models, which resulted in exaggerated diagnostic performance and were not applicable to the biopsy‐naive population.^16^ Thus, in this study, we constructed and externally validated a novel nomogram incorporating PI‐RADS, PSAD plus age and provided a risk assessment tool using PSAD stratification to further optimize the early detection of csPCa and reduce unnecessary biopsies in biopsy‐naive population who underwent transrectal ultrasound‐guided transperineal cognitive biopsy.

## 2 MATERIALS AND METHODS

### 2.1 Patient population and study design

A total of 932 consecutive patients who initially received mpMRI before biopsy decision and subsequently underwent transrectal ultrasound‐guided transperineal cognitive biopsy at the First Affiliated Hospital of USTC (*n* = 503, between June 2018 and June 2021 and *n* = 246, between July 2021 and February 2023) and the Second Affiliated Hospital of Nanjing Medical University (*n* = 183, between July 2021 and February 2023) were enrolled in this multi‐institutional retrospective study (Figure S1). Of these, the 503 participants were regarded as the development cohort to construct the nomogram and conduct internal validation. External validation was performed on 246 and 183 participants, respectively.

This study was approved by the Institutional Ethics Committee of the First Affiliated Hospital of USTC (No. 20170179) and the Second Affiliated Hospital of Nanjing Medical University (No. 2020KY101). Written informed consent was obtained from all included patients.

### 2.2 MRI protocol and prostate biopsy

mpMRI was achieved using 3.0 Tesla scanners with a body array coil before prostate biopsy and the entire imaging data were evaluated to obtain PI‐RADS v2 score corresponding to each patient by dedicated radiologists with at least 5 years of experience in urinary system mpMRI reading. The radiologists were blind to histopathological results and other clinicodemographic characteristics. Then, transrectal ultrasound‐guided transperineal cognitive biopsies were performed by two experienced urologists in each institution. Briefly, we made a minor modification to take 10 cores in the peripheral zone and 2 cores in the transition zone based on Meyer et al.'s pattern,^17^ and obtained extra cores of cognitive fusion target biopsy if mpMRI indicated any suspicious lesions. The primary endpoint was the detection of csPCa, which was defined as a Gleason score of 3 + 4 or higher.^18^

### 2.3 Data collection

Recorded clinical characteristics included age, body mass index (BMI), serum PSA, PV, PSAD, abnormal DRE, PI‐RADS score, the number of cores, and familial history of PCa. PV was measured by mpMRI with a traditional ellipsoid formula (maximum antero‐posterior dimension × maximum longitudinal dimension × maximum transverse dimension × 0.52), and PSAD referred to the ratio of total serum PSA to PV. The definition of abnormal DRE is the presence of induration, nodularity, significant asymmetry, or loss of anatomical landmarks of the prostate gland as identified by the screening clinicians in the outpatient department.^19^

### 2.4 Statistical analysis

Patients in each cohort were divided into csPCa and non‐csPCa subgroups on the basis of the post‐biopsy pathological diagnosis. The continuous variables were presented as mean ± standard deviation and the categorical variables were expressed as original data plus percentages. Independent sample *t*‐test, Mann–Whitney *U*‐tests, and chi‐squared test were used as suitable to assess differences between two subgroups.

Univariate and multivariate logistic regression analysis were performed to calculate the correlation between the covariate and dependent variables, videlicet odds ratio (OR) and 95% confidence interval (95% CI) and filtrate the independent predictors. Variables with statistical significance in multivariate logistic analysis were included to formulate our novel nomogram. Performance of this model was evaluated in the development cohort and two validation cohorts via the bootstrap method. Discrimination was quantified and visualized using the area under the curve (AUC) by the charting receiver operating characteristic (ROC) curve. Calibration plots were used to analyze the consistency between observed endpoints and predictions, which could be reflected by the unreliability test and Brier score. Furthermore, the net benefits at different threshold probabilities were measured through decision curve analysis (DCA).

Statistical analysis was performed by SPSS 26.0 statistical software (Armonk, NY, USA), R version 4.2.3 (Luntek Technology Ltd, NJ, USA), and MedCalc software 15.2 (MedCalc Software Ltd, Ostend, Belgium), and statistical significance was defined as *p* < 0.05.

## 3 RESULTS

### 3.1 Study cohort characteristics

Overall, a total of 503 cases were enrolled in the development cohort, 246 and 183 cases were included in validation cohort 1 and validation cohort 2, respectively (Table 1). The detection rates of csPCa were 173/503 (34.39%), 102/246 (41.46%), and 80/183 (43.72%) for the development cohort and two validation cohorts, respectively. The data of BMI, the number of cores, and familial history of PCa were not statistically significant between csPCa and non‐csPCa groups in three cohorts (*p* > 0.05). Conversely, patients with csPCa were older (in the development cohort and validation cohort 2), owned lower PV (in the development cohort and validation cohort 1), and had higher PSA, PSAD, larger proportion of abnormal DRE (in all cohorts).

**TABLE 1:** Baseline characteristics for 932 biopsy‐naive patients in the development cohort and validation cohorts.

Characteristics	Development cohort (*n* = 503)			Validation cohort 1 (*n* = 246)			Validation cohort 2 (*n* = 183)		
	csPCa (*n* = 173)	Non‐csPCa (*n* = 330)	*p* value	csPCa (*n* = 102)	Non‐csPCa (*n* = 144)	*p* value	csPCa (*n* = 80)	Non‐csPCa (*n* = 103)	*p* value
Age (years)	71.47 ± 6.75	67.12 ± 9.32	<0.001	69.72 ± 7.92	67.84 ± 9.07	0.094	71.93 ± 8.63	67.55 ± 7.88	<0.001
BMI (kg/m^2^)	22.18 ± 2.87	22.44 ± 2.76	0.321	22.38 ± 2.75	22.01 ± 2.94	0.319	22.04 ± 2.74	22.78 ± 2.87	0.079
PSA (ng/mL)	69.02 ± 66.32	17.02 ± 23.65	<0.001	79.87 ± 109.02	18.80 ± 21.96	<0.001	58.55 ± 77.87	11.51 ± 13.91	<0.001
PV (mL)	47.32 ± 25.81	61.82 ± 31.41	<0.001	41.22 ± 32.49	53.78 ± 27.37	0.001	48.03 ± 30.32	52.42 ± 31.28	0.342
PSAD (ng/mL^2^)	1.53 ± 1.29	0.34 ± 0.45	<0.001	2.07 ± 2.26	0.44 ± 0.61	<0.001	1.61 ± 3.18	0.30 ± 0.47	<0.001
Abnormal DRE (%)	110 (63.58)	174 (52.73)	0.020	83 (81.37)	63 (43.75)	<0.001	51 (63.75)	31 (30.10)	<0.001
PI‐RADS score (%)									
≤2	5 (2.89)	97 (29.39)		1 (0.98)	42 (29.17)		2 (2.50)	46 (44.66)	
3	15 (8.67)	137 (41.52)		12 (11.76)	55 (38.19)		16 (20.00)	24 (23.30)	
>3	153 (88.44)	96 (29.09)		89 (87.25)	47 (32.64)		62 (77.50)	33 (32.04)	
Number of cores	12.45 ± 4.15	13.12 ± 2.66	0.056	12.68 ± 2.30	12.27 ± 3.10	0.249	12.70 ± 3.62	12.50 ± 2.97	0.675
Family history of PCa (%)	2 (1.16)	3 (0.91)	0.791	3 (2.94)	1 (0.69)	0.170	2 (2.50)	2 (1.94)	0.798

Abbreviations: csPCa, clinically significant prostate cancer; BMI, body mass index; PSA, prostate‐specific antigen; PV, prostate volume; PSAD, prostate‐specific antigen density; DRE, digital rectal examination; PI‐RADS, Prostate Imaging‐Reporting and Data System.

### 3.2 Development of a novel nomogram and internal validation

In the development cohort, the univariate logistic regression analysis demonstrated that age, PSA, PV, PSAD, abnormal DRE, and PI‐RADS score were significant independent predictors of csPCa (Table 2). On multivariate logistic regression analysis, age (OR, 1.075; 95% CI, 1.036–1.114; *p* < 0.001), PSAD (OR, 6.003; 95% CI, 2.826–12.751; *p* < 0.001), and PI‐RADS score (OR, 3.419; 95% CI, 2.453–4.766; *p* < 0.001), but not PSA, PV, and abnormal DRE, were significantly associated with csPCa (Table 2). Besides, Table S1 showed the results of multivariate logistic regression analysis containing only age, PSAD, and PI‐RADS score, which is consistent with the statistical significance of Table 2.

**TABLE 2:** Univariate and multivariate regression analyses for predicting clinically significant prostate cancer.

Parameters	Univariate			Multivariate			
	OR	95% CI	*p* value	B	OR	95% CI	*p* value
Age	1.066	1.040–1.092	<0.001	0.072	1.075	1.036–1.114	<0.001
PSA	1.048	1.037–1.058	<0.001	0.008	1.008	0.996–1.020	0.197
PV	0.980	0.973–0.988	<0.001	−0.006	0.994	0.984–1.005	0.320
PSAD	18.483	10.406–32.827	<0.001	1.792	6.003	2.826–12.751	<0.001
Abnormal DRE	1.565	1.073–2.284	0.020	0.255	1.291	0.723–2.302	0.388
PI‐RADS score	4.926	3.720–6.523	<0.001	1.229	3.419	2.453–4.766	<0.001

Abbreviations: 95% CI, 95% confidence intervals; DRE, digital rectal examination; OR, odds ratio; PI‐RADS, Prostate Imaging‐Reporting and Data System; PSA, prostate‐specific antigen; PV, prostate volume; PSAD, prostate‐specific antigen density.

**FIGURE 1:**
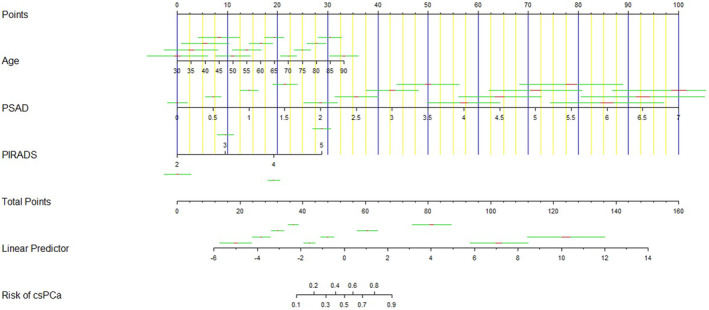
Novel nomogram predicting the probability of csPCa for biopsy‐naive patients based on the development cohort. PSAD, prostate‐specific antigen density; PIRADS, Prostate Imaging‐Reporting, and Data System; csPCa, clinically significant prostate cancer.

**FIGURE 2:**
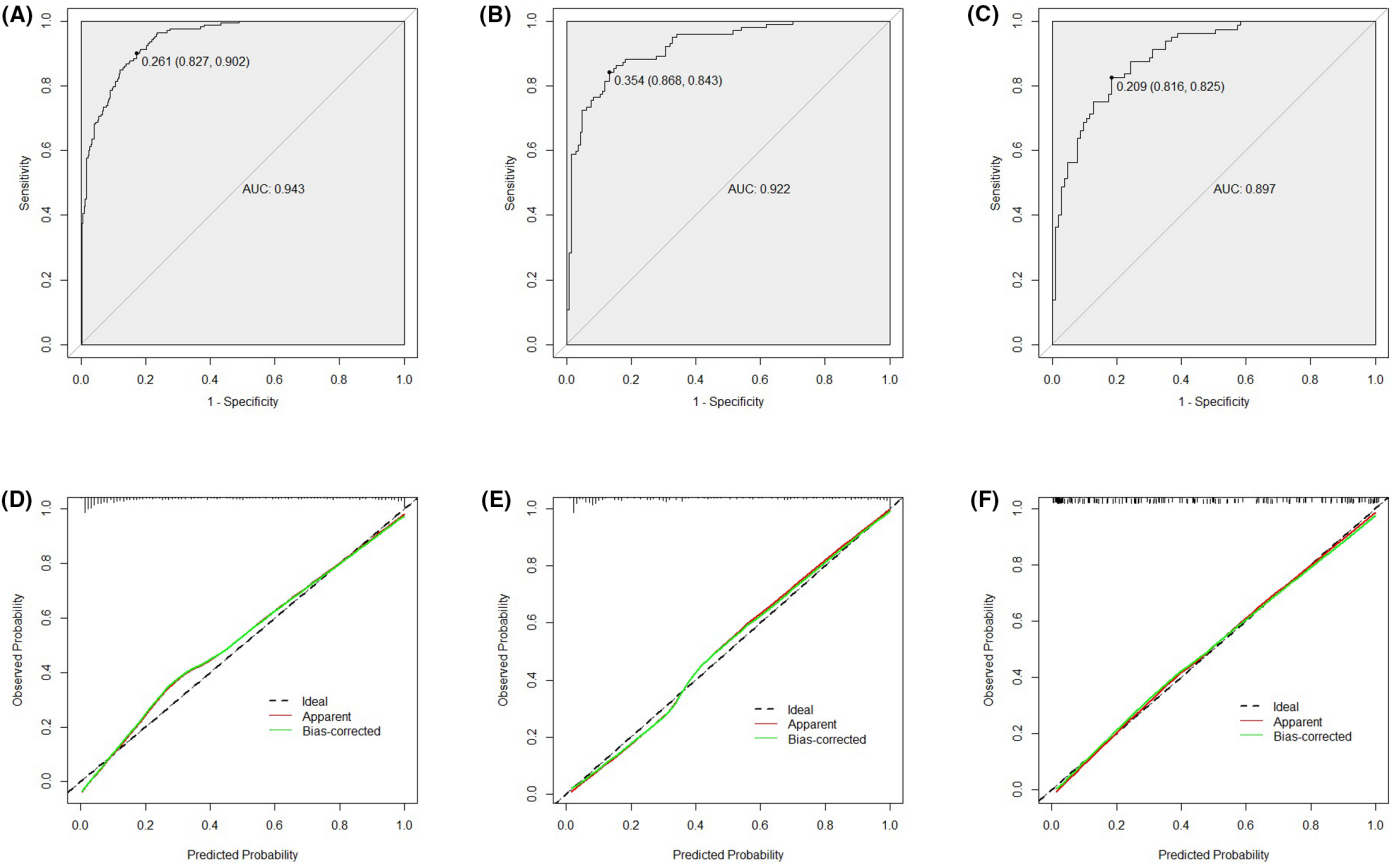
Discrimination and calibration of this model were internally and externally inspected. The ROC curves of the development cohort (A), validation cohort 1 (B), and validation cohort 2 (C). The calibration plots of development cohort (D), validation cohort 1 (E), and validation cohort 2 (F). The broken blue line represents the ideal model. Predicted probability is plotted on the x‐axis and observed probability is plotted on the y‐axis. ROC, receiver operating characteristic.

The constructed nomogram based on the multivariate model of Table S1 is illustrated in Figure 1 to predict csPCa in biopsy‐naive patients. As shown in Table S2, our novel model achieved the highest AUC (0.943, Figure 2A), specificity (90.2%), positive predictive value (PPV, 74.9%), and accuracy (86.7%) on internal validation compared to the individual covariate (age, PSAD, or PI‐RADS score), Model 1 (fitted by age and PSAD), Model 2 (fitted by age and PI‐RADS score), and Model 3 (fitted by PSAD and PI‐RADS score). The Spiegelhalter *Z*‐statistic *p*‐value of unreliability test was 0.713 and the Brier score was 0.092 (Figure 2D), which indicated a good agreement between prediction and observation in the development cohort.

### 3.3 External validation and decision curve analysis

To evaluate the performance of this model in two validation cohorts, ROC curves and calibration plots were also administered to estimate discrimination and calibration. As illustrated in Figure 2B,C, the AUCs for the validation cohort 1 and cohort 2 were 0.922 and 0.897, respectively. Both calibration plots (Figure 2E,F) had excellent performance, manifesting as low Brier scores (0.102 and 0.133, respectively) and insignificant Spiegelhalter *Z*‐statistic *p*‐values (0.490 and 0.859, respectively), which suggested minute differences between predicted and observed probabilities.

**FIGURE 3:**
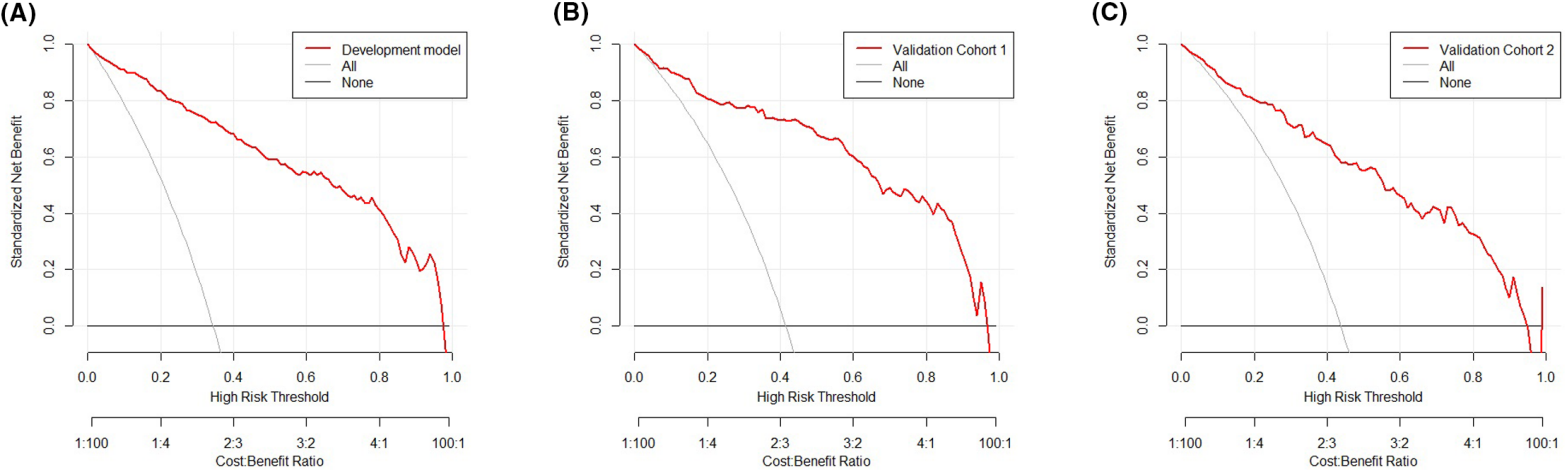
Decision curve analysis of clinical use of this nomogram in all three cohorts: (A) development cohort, (B) validation cohort 1, and (C) and validation cohort 2.

With the elevation in risk thresholds, net benefits were found to be superior to those of the “treat all” and “treat none” approaches in all three cohorts (Figure 3), indicating the clinical usefulness could be markedly improved by applying this nomogram in biopsy‐naive men.

### 3.4 Performance under different PSAD cutoffs

As PSAD played an important role (high OR value) in csPCa prediction in our nomogram and PI‐ RADS v2 score 3 lesions are generally considered to be equivocal for ascertaining csPCa, these patients were stratified by PSAD value to groups: PSAD <0.2 versus ≥0.2 ng/ml^2^, and <0.3 versus ≥0.3 ng/ml^2^ (Table 3). Of the 152 involved patients, csPCa was excluded in 98.51% of men for PSAD <0.2 ng/ml^2^, compared to 81.18% for PSAD ≥0.2 ng/ml^2^. Similarly, the probability of not harboring csPCa increased to 96.81% for PSAD <0.3 ng/ml^2^, compared to 75.86% for PSAD ≥0.3 ng/ml^2^. Other performance indicators, including sensitivity (94.1% and 82.4%, respectively), specificity (48.9% and 67.4%, respectively), NPV (98.5% and 96.8%, respectively), PPV (18.8% and 24.1%, respectively), and accuracy (53.9% and 69.1%, respectively) are also listed in Table 3.

**TABLE 3:** Performance features of different PSAD thresholds for biopsy‐naive patients with PI‐RADS 3 lesions.

	csPCa (%)	Sensitivity (%)	Specificity (%)	NPV (%)	PPV (%)	Accuracy (%)
PSAD < 0.2 (ng/mL^2^)	1/67 (1.5)					
PSAD ≥ 0.2 (ng/mL^2^)	16/85 (18.8)	94.1	48.9	98.5	18.8	53.9
PSAD < 0.3 (ng/mL^2^)	3/94 (3.2)					
PSAD ≥ 0.3 (ng/mL^2^)	14/58 (24.1)	82.4	67.4	96.8	24.1	69.1

Abbreviations: csPCa, clinically significant prostate cancer; NPV, negative predictive value; PI‐RADS, Prostate Imaging‐Reporting and Data System; PPV, positive predictive value; PSAD, prostate‐specific antigen density.

## 4 DISCUSSION

The detection rate of csPCa in this multicenter study was approximately ranging from 35% to 45% after transrectal ultrasound‐guided transperineal cognitive biopsy, which was slightly higher than 30% reported previously and parallels with the published data of roughly 40% for targeted biopsy.^20^, ^21^, ^22^ Subsequent univariate and multivariate logistic analysis revealed that increasing age, a raised elevated PSAD level, and PI‐RADS score were closely related to csPCa. Therefore, we applied these independent predictors to a multivariate model, with ROC curves, calibration plots, and decision curve analyses on internal and external validation evidencing that this novel nomogram could improve the diagnostic performance of csPCa and patients with PI‐RADS 3 lesions plus PSAD <0.2 ng/ml^2^ held a high likelihood of not harboring csPCa.

The UK National Screening Committee criteria declared that an ideal screening program should identify an acknowledged cutoff level to trigger a subsequent test. Despite numerous Chinese urologists standardizing PSA >4 ng/mL as an abnormal value according to the China guideline, the Prostate Cancer Prevention Trial particularly stated that the sensitivity of PSA for Gleason score ≥3 + 4 was 58% when the PSA threshold was 3 ng/mL and the threshold needed to be lowered to at least 1.6 ng/mL to achieve an acceptable 84% sensitivity,^12^ which means a suitable PSA cutoff level cannot be agreed as a hazard of missing csPCa at all levels of serum PSA. As for MRI, a randomized, noninferiority trial found that csPCa was detected more frequently in biopsy‐naive men undergoing MRI with or without targeted biopsy,^10^ whereas a Cochrane meta‐analysis noted that MRI was characterized with a mediocre pooled specificity of 0.37 (95% CI: 0.29–0.46) for International Society of Urological Pathology Grade 2 or higher PCa and a pooled detection ratio of only 1.05 (95% CI: 0.95 to 1.16) in patients who were biopsy‐naive.^23^ In anticipation of this inconsistent evidence, international guidelines recommended performing mpMRI before prostate biopsy in biopsy‐naive patients rather than as a screening tool.^24^, ^25^ However, mpMRI combined with other clinical variables and/or some advanced biomarkers may be practicable in PCa screening, for instance, Nordström et al.^26^ utilized the Stockholm3 test plus MRI to detect fewer low‐grade cancers and more csPCa compared with PSA‐based screening and these encouraging results prompted us to develop an appropriate prediction model to help Chinese clinicians in the early detection of csPCa.

Nomograms provide a method to orchestrate several parameters to obtain desired outcomes in different fields. Over half of the radiation oncologists and urologists reported employing PCa nomograms^27^ and the European Association of Urology guidelines strongly recommended using nomograms to identify the risk of patients performing extended lymph node dissections.^24^ Unfortunately, there is to our knowledge no extensively used and recognized nomograms to appraise the risk of csPCa in cancer screening, especially in China. In 2016, the Chinese Prostate Cancer Consortium Risk Calculator (CPCC‐RC) had been established to forecast the possibility of high‐grade PCa, which included age, PV, PSA, free PSA ratio, and DRE but without mpMRI and had an AUC of 0.826.^28^ Furthermore, Falagario et al. reported a prediction model composed of PSAD and mpMRI, manifesting as a great sensitivity of 96.70% but a poor specificity of 30.10%.^29^ In the present study, we introduced a novel nomogram with superior AUCs of 0.943 in the development cohort and ≥0.897 in the two validation cohorts, and higher sensitivity and specificity for csPCa (82.7% and 90.2%, respectively), which could reduce unnecessary biopsies and alleviate the burden of overdiagnosis and overtreatment, such as radical prostatectomy, androgen‐deprivation therapy or radiotherapy. In addition to mpMRI and PSAD, age was also determined as a significant predictor of csPCa in this model. We believed this result might be related to the lack of attention to cancer screening in China and an Asian autopsy study found the prevalence of latent PCa was about 10% in men aged 50s and increased to over 40% in their 80s,^30^ which indicated the prevalence of PCa increased with age.

Some published nomograms enrolled both prior negative biopsy and biopsy‐naive patients, which might result in the inapplicability of their models for the latter because the detection rate of csPCa is markedly lower in the prior negative biopsy setting in comparison with biopsy‐naive patients,^16^ especially in men with debatable PI‐RADS 3 lesions. Although mainstream guidelines moderately recommend performing a systematic biopsy and/or targeted biopsy for biopsy‐naive patients with PI‐RADS 3 lesions,^24^, ^25^ whereas the updated PI‐RADS v2 considered the option of leaving out any prostate biopsy if the patient was not at high risk of csPCa,^31^ which could be calculated by PSAD. Actually, the use of PSAD for enhanced risk stratification to avoid unnecessary biopsy has been applicable for the diagnostic evaluation of biopsy‐naive men. Schoots et al. categorized the included population into different PSAD risk groups: <0.10, 0.10–0.15, 0.15–0.20, and >0.20 ng/ml^2^, and the biopsy actions for patients with PI‐RADS 3 lesions were no biopsy, consider biopsy, highly consider biopsy, and perform biopsy, corresponding to aforementioned four PSAD ranges, respectively, and they believed this risk‐adapted matrix table based on PSAD and prostate MRI could help guide biopsy‐decision management.^32^ In the present analysis, the possibility of excluding csPCa was 98.51% for patients with PSAD <0.2 ng/ml^2^ and PI‐RADS 3 lesions, scilicet 44.08% (67/152) and 6.58% (10/152) of biopsy‐naive patients could refrain from instant biopsies and overdiagnosis of clinically insignificant PCa, respectively. These data could assist urologists in communicating with patients to make decisions together on safely omitting biopsies and proceeding with active surveillance in case of equivocal mpMRI findings and low potentiality of csPCa. Additionally, another important role of MRI images is to guide the later treatment, and external beam radiation therapy and brachytherapy are also currently widely recognized as the alternative treatment approach for patients diagnosed with intermediate‐risk or high‐risk diseases aside from radical prostatectomy. The hybrid systems for magnetic resonance guided radiotherapy combine MRI‐scanners with linear accelerators can minutely contour the lesions and allow for soft‐tissue tracking and gating during therapy.^33^

Recently, the applications of machine learning, as a subfield of artificial intelligence, are rapidly developing in PCa due to the numerous technological domains involved in the diagnosis, prognosis, and treatment. The first artificial intelligence‐based pathology solution for prostate biopsy has been approved by the United States Food and Drug Administration, which enhanced the observation and interpretation of low‐level image analysis tasks and high‐level inference and prediction assignments in clinical practice.^34^ A latest study of 1130 consecutive patients from a prospective database even demonstrated that the supervised machine learning algorithms could firmly predict biochemical recurrence after radical prostatectomy and surpass the conventional nomograms.^35^ Nevertheless, the development, operation, and regulation of artificial intelligence‐based procedures require abundant clinical information and the collaboration of clinicians (such as urologists, radiologists, and pathologists), programmers, and engineers.^36^ Hence, our further research will focus on satisfactorily integrating the advantages of computer and human being and we deem this burgeoning concept may revolutionize urology.

There are several notable limitations in our study. First, despite consecutive patients were enrolled in each cohort, incomplete data (such as the lack of comorbidity and lower urinary tract symptoms) and selection bias are potentially present in our study due to the deficiency inherent to a retrospective analysis. Second, the collected data were derived from two general hospitals in eastern China, which might limit its generalizability and should be cautious about popularizing this model to other countries and populations because of the diverse disease prevalence, image quality, and experience of the radiologist. Third, the ineffaceable subjectivity of DRE and interobserver variability in image reading may affect the existing performances of abnormal DRE and PI‐RADS score in logistic regression analysis. Lastly, we included only patients who underwent systematic biopsy with cognitive fusion biopsy and lacked the clinical data on MRI‐targeted biopsy.

## 5 CONCLUSIONS

Age, PSAD, and PI‐RADS v2 score were identified as significant independent predictors of csPCa and used to construct a robust nomogram in this study. Adoption of this nomogram in cancer screening could optimize patient selection for prostate biopsy and biopsy‐naive patients with PSAD <0.2 ng/ml^2^ plus PI‐RADS 3 lesions could safely forego immediate biopsy because of the low probability of harboring csPCa (1.49%).

## AUTHOR CONTRIBUTIONS

**Qingyu Ge:** Conceptualization (lead); formal analysis (equal); methodology (lead); software (equal); validation (equal); visualization (equal); writing – original draft (equal). **Sicong Zhang:** Conceptualization (equal); data curation (equal); formal analysis (equal); investigation (equal); software (equal). **Hewei Xu:** Conceptualization (equal); investigation (equal); methodology (equal); resources (equal); software (equal); visualization (equal). **Junjie Zhang:** Data curation (equal); formal analysis (equal); resources (equal); software (equal). **Zongyao Fan:** Formal analysis (equal); investigation (equal); validation (equal). **Weilong Li:** Data curation (equal); investigation (equal); resources (equal). **Deyun Shen:** Data curation (equal); formal analysis (equal); investigation (equal). **Jun Xiao:** Project administration (equal); visualization (equal); writing – review and editing (equal). **Zhongqing Wei:** Conceptualization (equal); funding acquisition (equal); project administration (equal); supervision (equal); writing – review and editing (equal).

## FUNDING INFORMATION

This work was funded by the National Key Technology R&D Program of China (No. 2018YFC2002204).

## CONFLICT OF INTEREST STATEMENT

The authors made no disclosures.

## ETHICS STATEMENT

This study was approved by the Institutional Ethics Committee of the First Affiliated Hospital of USTC (No. 20170179) and the Second Affiliated Hospital of Nanjing Medical University (No. 2020KY101).

## PATIENT CONSENT STATEMENT

Informed consent was obtained from all eligible patients.

## Supporting information


Figure S1.

Table S1.

Table S2.

## Data Availability

The data that support the findings of this study are available from the corresponding author upon reasonable request.
