# Supplementary figures and images for: Development and validation of a novel nomogram predicting clinically significant prostate cancer in biopsy‐naive men based on multi‐institutional analysis
Source: Cancer Med. 2023 Nov 28;12(24):21820–9. doi: 10.1002/cam4.6750 (PMC10757090; doi:10.1002/cam4.6750)

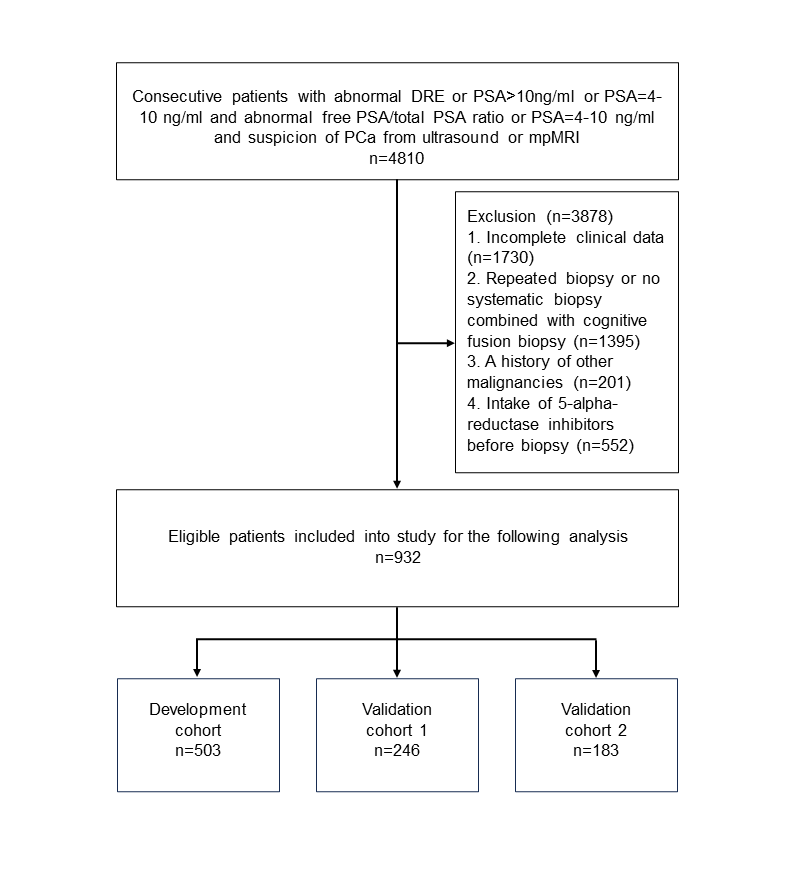

Supplement: Supplementary file 1 — Figure S1. [file CAM4-12-21820-s002.tif]
