# Supplementary material for: Development and validation of a novel nomogram predicting clinically significant prostate cancer in biopsy‐naive men based on multi‐institutional analysis
Source: Cancer Med. 2023 Nov 28;12(24):21820–9. doi: 10.1002/cam4.6750 (PMC10757090; doi:10.1002/cam4.6750)
Supplement: Supplementary file 2 — Table S1. [file CAM4-12-21820-s001.docx]

**Table S1** Detailed results of multivariate logistic regression analysis for detecting csPCa

| Indicator | B | S | Wals | OR | 95%CI | *p* value |
| --- | --- | --- | --- | --- | --- | --- |
| Age | 0.073 | 0.018 | 15.937 | 1.076 | 1.038-1.116 | <0.001 |
| PSAD | 2.145 | 0.310 | 47.874 | 8.539 | 4.651-15.676 | <0.001 |
| PI-RADS score | 1.276 | 0.168 | 57.828 | 3.582 | 2.578-4.977 | <0.001 |
| Constant | -12.048 | 1.558 | 59.837 | 0.000 | - | <0.001 |

csPCa = clinically significant prostate cancer; PSAD = prostate-specific antigen density; PI-RADS = Prostate Imaging-Reporting and Data System; OR = odds ratio; 95% CI = 95% confidence intervals.
