# Supplementary material for: Development and validation of a novel nomogram predicting clinically significant prostate cancer in biopsy‐naive men based on multi‐institutional analysis
Source: Cancer Med. 2023 Nov 28;12(24):21820–9. doi: 10.1002/cam4.6750 (PMC10757090; doi:10.1002/cam4.6750)
Supplement: Supplementary file 3 — Table S2. [file CAM4-12-21820-s003.docx]

| Indicator | Age | PSAD | PI-RADS | Model 1 |  | Model 2 | Model 3 |  | Development model |
| --- | --- | --- | --- | --- | --- | --- | --- | --- | --- |
| AUC | 0.639 | 0.918 | 0.844 | 0.917 |  | 0.872 | 0.940 |  | 0.943 |
| Sensitivity (%) | 78.6 | 86.1 | 88.4 | 90.7 |  | 85.6 | 94.8 |  | 82.7 |
| Specificity (%) | 45.2 | 81.5 | 70.9 | 79.1 |  | 77.9 | 81.8 |  | 90.2 |
| PPV (%) | 42.9 | 71.0 | 61.4 | 69.5 |  | 67.0 | 73.2 |  | 74.9 |
| NPV (%) | 80.1 | 91.8 | 92.1 | 94.2 |  | 91.1 | 96.8 |  | 94.9 |
| Accuracy (%) | 56.7 | 82.9 | 76.9 | 85.1 |  | 80.1 | 86.3 |  | 86.7 |

**Table S2** Performance characteristics of individual predictor and all available models

PSAD = prostate-specific antigen density; PI-RADS = Prostate Imaging-Reporting and Data System;

AUC = area under the curve; PPV = positive predictive value; NPV = negative predictive value.
